# Ultrasound-Guided Intrathecal Baclofen Pump Refilling Method for Management of Spasticity in a Complex Clinical Case

**DOI:** 10.7759/cureus.31537

**Published:** 2022-11-15

**Authors:** Sérgio Pinho, Anabela Ferreira, Duarte Calado, Madjer Hatia, Filipa Faria

**Affiliations:** 1 Physical Medicine and Rehabilitation, Centro Hospitalar Lisboa Ocidental, Lisboa, PRT; 2 Physical Medicine and Rehabilitation, Centro de Medicina de Reabilitação de Alcoitão, Lisboa, PRT

**Keywords:** ultrasound guidance, pump refill, intrathecal baclofen therapy, baclofen, spasticity

## Abstract

Intrathecal baclofen (ITB) administration is a common method in managing spasticity. The location of the reservoir fill port (RFP) is identified manually in most cases. However, it can be difficult due to a variety of factors, such as the formation of excess subcutaneous cellular tissue and scar formation overlying the RFP and rotation or inversion of the pump. Consequently, multiple failed attempts accessing the reservoir increases pain and risk of fatal complications (e.g., infection and withdrawal syndrome from pocket filling). We describe a successful ultrasound-guided pump refilling case after multiple failed attempts by the conventional method. This groundbreaking instrument assists this minimally invasive procedure while limiting iatrogenic injury in the treatment of spasticity. The presentation of this case shows the utility of ultrasound as an important tool to guide the procedure and prevent adverse events in a spasticity management consult.

## Introduction

Spasticity is an upper motor neuron sign characterized by an increase in velocity-dependent muscle tone [1]. Although it may sometimes be helpful in certain activities of daily living (e.g., transfers, ambulation, and postural control), spasticity is mainly associated with negative characteristics, such as loss of motor control, painful spasms, and contractures, which often leads to functional disability that compromises independence, participation, and quality of life [2,3].

Baclofen is a gamma-aminobutyric acid (GABA) type B receptor agonist in the spinal cord. This drug acts at the presynaptic level, inhibiting the release of excitatory neurotransmitters, and postsynaptically, making cell depolarization difficult. It is used to reduce spasticity, clonus, spasm frequency, and neuropathic pain [4].

Intrathecal baclofen (ITB) administration is a therapeutical option in spasticity management of chronic patients in whom oral baclofen and focal therapy with botulinum toxin are not effective in reducing muscle tone or side effects are poorly tolerated [3]. It is commonly administered by a programmable pump, surgically implanted in the abdominal wall, and delivered through a catheter positioned in the intrathecal space [5].

An ITB pump needs to be refilled periodically to control muscle tone and avoid the onset of withdrawal syndrome and its harmful consequences. Refilling involves inserting a needle into the pump access port, puncturing the silicone septum of the reservoir, entering the RFP, and then injecting the drug into it. It is recommended to use a template to locate the puncture site and access the refill port, in which the edges of the pump must be aligned using the palpatory method. However, this method can be difficult for various reasons, such as formation of a thick layer of subcutaneous cellular tissue due to weight gain after implantation of the pump, hypermobility of the pump, scar formation above the pump surface, patient positioning, or inexperienced clinician. This makes it challenging to guide the needle correctly leading to multiple punctures, increasing patient discomfort and risk of complications (e.g., infection and pocket fills). There is also a risk of missing the target and infiltrating the subcutaneous cellular tissue, with the possibility of drug overdosing or underdosing, which can generate a potentially fatal complication [6]. The accuracy of this blind technique appears to be poor when compared to other guided methods [7]. Thus, we report the case of an elderly woman with spinal cord injury (SCI) who underwent an ultrasound-guided pump refilling after multiple unsuccessful attempts by the conventional method.

## Case presentation

The patient was a 72-year-old female who had a history of a traumatic SCI that resulted in spastic incomplete paraplegia (American Spinal Injury Association Impairment Scale B) with the level of injury T11, undergoing physical therapy on a regular basis for 21 years since its onset. It was a severe case of spasticity in the lower limbs (grade 4 in dorsiflexors and plantar flexors and grade 3 in the other remaining muscle groups according to the modified Ashworth scale) that required a lot of assistance in activities of daily living.

**Figure 1:**
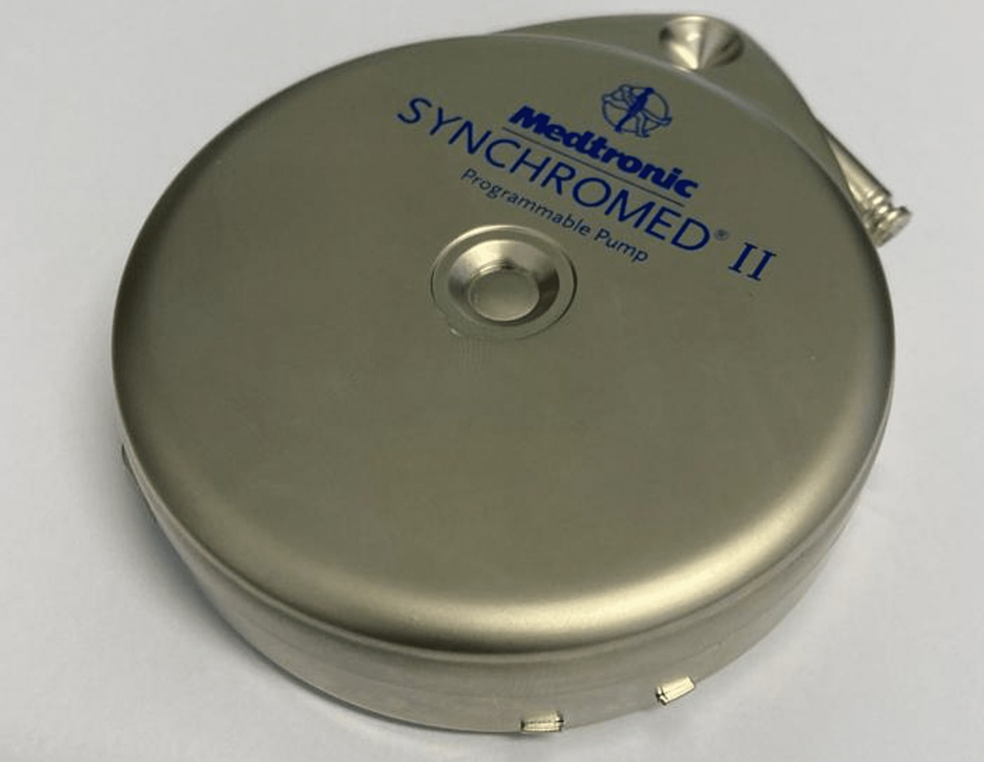
Medtronic SynchroMedTM II intrathecal pump.

As she remained refractory to oral therapy with baclofen (150 mg per day), she was referred to Neurosurgery to perform the implantation of an ITB pump. After a good response to an ITB infusion trial in 2005, she underwent Medtronic SynchroMedTM II intrathecal pump (Figure 1) implantation surgery. Over the years, this dose was progressively increased to the current dosage of 379.8 µg/day. In addition, the patient also progressively gained weight, increasing 10 kg in 17 years after the first pump was placed.

Re-evaluation was done every 4 to 5 months for periodic refilling of baclofen. The refilling process proved to be increasingly challenging, because of significant subcutaneous fat overlying the pump, where the pump could not be easily palpated, requiring multiple attempts to locate its entry point. These refills were time-consuming and stressful for both the patient and the physician.

Procedure description

**Figure 2:**
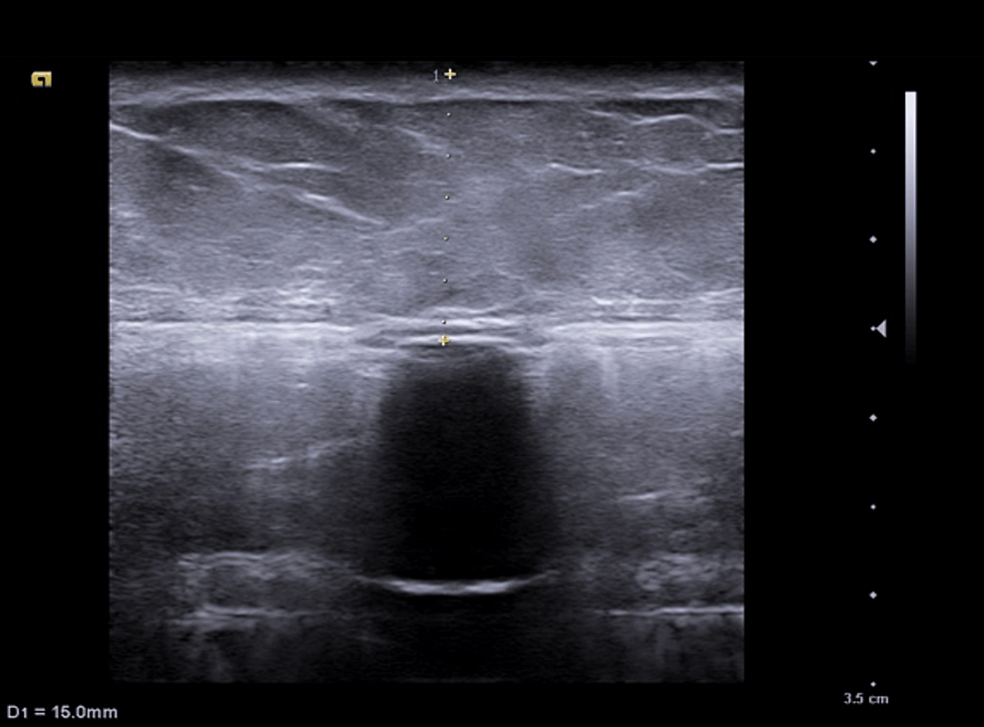
Ultrasound imaging of ITB pump. The surface of the intrathecal pump is identified by a hyperechoic line with acoustic shadowing. A hypoechoic shadow represents the RFP deep into the silicone membrane. Distance skin–pump entry (D1): 15 mm. ITB: intrathecal baclofen; RFP: reservoir fill port

We used a Siemens Acuson NX3 ultrasound device with a 10 MHz high-frequency linear array transducer to locate the RFP of the ITB pump. A well-defined hyperechoic line with acoustic shadowing was identified as the surface of the metallic pump. The silicone septum of the RFP was identified as a hypoechoic area, surrounded by the hyperechoic line. Subcutaneous fat overlying the pump was measured, presenting 15 mm of thickness (Figure 2). The transducer was moved across the pump in two perpendicular planes to center the port on the ultrasound screen.

**Figure 3:**
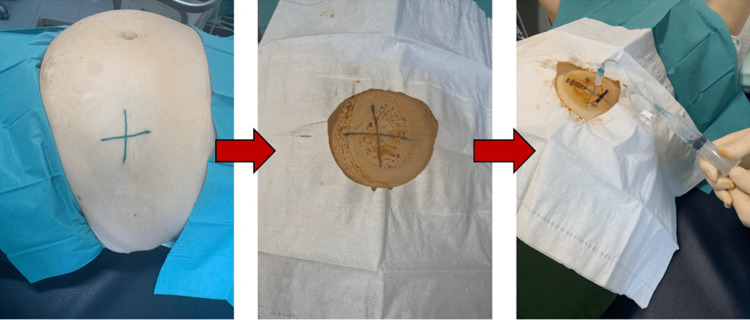
Technical procedure for refilling the baclofen pump after marking the puncture site using ultrasonography.

Using a green dermographic pen, we marked the perpendicular lines in which the intersection point corresponded to the puncture site (Figure 3). Then, the region was disinfected with iodopovidone. When using the template, we verified that its central region didn’t correspond to the intersection point outlined previously by ultrasound. Subsequently, the refill procedure was completed using the 22G needle included in the Medtronic SynchroMedTM II kit. After refilling, an ultrasound was used to ensure that no subcutaneous or pump-pocket fluid was present.

## Discussion

Ultrasound is progressively gaining value in many musculoskeletal and neurologic disorders. It is convenient, inexpensive, non-invasive, and does not expose the patient to radiation. The real-time capability of ultrasonography provides access to anatomic structures and assistance with minimally invasive procedures, limiting iatrogenic injury, and other intervention-related complications [8]. Therefore, it is considered an important tool for many physicians who specialize in the management of spasticity.

Refilling baclofen pumps in difficult cases has become considerably easier and safer due to the use of ultrasound guidance [9]. This allows for the visualization of the reservoir and ensures the higher success of refill in pumps placed deeper than 10 mm [10]. The RFP can be clearly located as a hypoechoic rectangle between the hyperechoic lines of the metallic pump. It has been proven that the ultrasound-guided ITB pump refill method reduces procedure-related pain and increases patient satisfaction, even though it lengthened the duration of refills compared to the template-guided method [11].

There are two types of ultrasound-guided techniques that have been described for accessing the RFP: the indirect and direct methods. The indirect method, explained in this clinical case, consists of using ultrasound to mark the RFP puncture site and, after the refill, scan for eventual subcutaneous injection [12]. The direct method is a real-time visualization of the needle accessing the silicone septum of the RFP, commonly using an out-of-plane technique [13]. For the direct method we recommend the usage of an ultrasound probe protective sterilized cover made in latex in the detriment of polyethylene or polyurethane covers. This method guarantees that there is no need for the use of ultrasound gel as an interface between the latex and the skin of the patient, once the safety of the ultrasound gel if introduced into the reservoir or intrathecally, has not been established [14]. Furthermore, gel between the probe and its cover is needed in order to transmit ultrasound waves to reach the anatomical structures and render an image more accurately.

One study has implemented the color Doppler mode in the ultrasound-guided refilling process by injecting a minimal volume of baclofen, providing a column-shaped jet that allowed the understanding of whether the product was being distributed intra or extra-pump. Alternatively, aspiration of the pump content will provide the same effect. This ensures another safety marker for the procedure and enhances the accuracy of this technique, but it has only been described in a cadaveric model requiring further clinical investigation [15].

Ultrasound has also been demonstrated to be helpful in the presence of seroma and in identifying pocket fills [14,16]. In addition, ultrasound allows for the identification of ITB pumps that have flipped within the pump pocket as surface scanning will only reveal a solid hyperechoic outline [15].

## Conclusions

Ultrasound-guided access to the entry point of ITB pumps is a safe and easy method for refilling the reservoir in technically challenging cases. It may facilitate the procedure and prevent complications related to multiple and/or incorrect subcutaneous injections compared to the conventional technique.

As new applications and the accessibility of ultrasound increase worldwide, we expect that, in a near future, this tool will be available in most hospitals and spasticity clinics ensuring the procedure success, reduction of iatrogenic injury, and consequent improvement in spastic patient satisfaction.

## References

[1] Bhimani R, Anderson L (2014). Clinical understanding of spasticity: implications for practice. Rehabil Res Pract.

[2] Brainin M, Norrving B, Sunnerhagen KS (2011). Poststroke chronic disease management: towards improved identification and interventions for poststroke spasticity-related complications. Int J Stroke.

[3] Ertzgaard P, Campo C, Calabrese A (2017). Efficacy and safety of oral baclofen in the management of spasticity: a rationale for intrathecal baclofen. J Rehabil Med.

[4] Bottros MM, Christo PJ (2014). Current perspectives on intrathecal drug delivery. J Pain Res.

[5] Francisco GE, Saulino MF, Yablon SA, Turner M (2009). Intrathecal baclofen therapy: an update. PM R.

[6] Caruso P, Mazzon G, Sarra VM, Tacconi L, Manganotti P (2018). The use ultrasound guided for refilling intrathecal baclofene pump in complicated clinical cases: a practical approach. J Clin Neurosci.

[7] Maino P, Koetsier E, Perez RSGM (2015). The accuracy of template-guided refill technique of intrathecal pumps controlled by fluoroscopy: an observational study. Neuromodulation.

[8] Özçakar L, Tok F, De Muynck M, Vanderstraeten G (2012). Musculoskeletal ultrasonography in physical and rehabilitation medicine. J Rehabil Med.

[9] Aras B, Kesikburun S, Adıgüzel E, Yılmaz B (2016). Ultrasound guidance for intrathecal baclofen pump refill. Turk J Phys Med Rehabil.

[10] Matthys C, Jacobs M, Rossat J, Perruchoud C (2020). Accuracy of template versus ultrasound identification of the reservoir access port of intrathecal drug delivery system. Neuromodulation.

[11] Singa RM, Buvanendran A, McCarthy RJ (2020). A comparison of refill procedures and patient outcomes following ultrasound-guided and template-guided intrathecal drug delivery systems with recessed ports. Neuromodulation.

[12] Maneyapanda MB, Chang Chien GC, Mattie R, Amorapanth P, Reger C, McCormick ZL (2016). Ultrasound guidance for technically challenging intrathecal baclofen pump refill: three cases and procedure description. Am J Phys Med Rehabil.

[13] Hurdle MFB, Locketz AJ, Smith J (2007). A technique for ultrasound-guided intrathecal drug-delivery system refills. Am J Phys Med Rehabil.

[14] Shankar H (2009). Ultrasound-guided localization of difficult-to-access refill port of the intrathecal pump reservoir. Neuromodulation.

[15] Gofeld M, McQueen CK (2011). Ultrasound-guided intrathecal pump access and prevention of the pocket fill. Pain Med.

[16] Peccora CD, Ross EL, Hanna GM (2013). Aberrant intrathecal pump refill: ultrasound-guided aspiration of a substantial quantity of subcutaneous hydromorphone. Reg Anesth Pain Med.

